# The Gifted Outlier in Academic Medicine: Toward a Balanced Framework for Recognizing Diverse Excellence

**DOI:** 10.7759/cureus.99399

**Published:** 2025-12-16

**Authors:** Dimitrios Moris

**Affiliations:** 1 Department of Surgery, MedStar Georgetown University Hospital, Durham, USA; 2 School of Medicine, European University of Cyprus, Nicosia, CYP

**Keywords:** academic medicine, institutional culture, outliers, physician-scientist, prolific authorship

## Abstract

Academic medicine increasingly evaluates faculty through quantifiable productivity metrics, including publication counts, citation indices, and grant funding. Within this environment, a small subset of clinicians and physician-scientists produce scholarly output at rates far exceeding disciplinary norms. These highly productive individuals can accelerate innovation, attract trainees, and elevate institutional reputation. Yet extreme productivity also raises legitimate concerns about authorship practices, data oversight, research integrity, and equitable resource allocation. This editorial proposes a balanced framework for understanding academic “outliers,” offering an operational definition, examining structural and individual factors that drive exceptional productivity, and outlining institutional safeguards that ensure rigor, transparency, and fairness. Recognizing differentiated forms of excellence-clinical, educational, scientific, and systems improvement can help academic medicine support high-performing faculty without compromising integrity or equity.

## Editorial

Academic medicine depends on faculty who contribute to clinical care, scientific discovery, education, and system innovation. Faculty evaluation frameworks, however, often compress these diverse activities into a limited set of quantitative metrics. In such an environment, some faculty members produce research at a pace that far exceeds disciplinary norms. These individuals, often labeled “outliers,” can shape clinical practice, generate new scientific directions, and serve as intellectual anchors within their institutions. Yet their presence also exposes tensions in how academic medicine evaluates productivity, ensures research integrity, and distributes institutional resources.

A meaningful discussion of outliers requires definitional clarity. For this editorial, an outlier is defined as a faculty member whose scholarly productivity ranks within the top approximately 5% of their field when normalized for team size, disciplinary publication norms, and authorship role, sustained for at least three consecutive years, and accompanied by evidence of substantive intellectual contribution and supervisory responsibility. This definition aligns with literature demonstrating that productivity varies widely by discipline and that raw counts do not sufficiently reflect actual contribution [1,2] (Table 1).

**Table 1:** Characteristics of academic-medicine outliers

Dimension	Candidate Characteristics	Notes
Productivity	Top ~5% field-normalized output	Adjusted for authorship role and disciplinary norms
Authorship	Documented, substantive contributions	CRediT taxonomy recommended
Funding	Sustained success in grants or team support	Reflects structural opportunity
Mentorship	Strong record of trainee development	Evidence from evaluations and career outcomes
Team Structure	Access to collaborative networks	Major determinant of the scale of output

The interplay of capability, opportunity, and structure and legitimate research-integrity considerations

High productivity emerges from an interaction of individual capability, structural support, and cumulative advantage. Research on expertise and performance consistently emphasizes that deliberate practice, mentorship, opportunity structures, and institutional resources play central roles in achievement [3-6]. Motivational factors, including goal orientation and intrinsic interest, also influence scholarly output, but these factors do not operate in isolation. Protected research time, robust research infrastructure, skilled statistical and project-management support, and access to collaborative networks are frequently necessary conditions for sustained productivity. Sociological work highlights the “Matthew effect,” whereby individuals who secure early mentorship, prestigious posts, or strong networks accumulate opportunity and visibility, further amplifying productivity over time [7]. This interplay suggests that exceptional output should not be framed as a product of innate giftedness but rather as an emergent property of capability interacting with opportunity, resources, and environment. At the same time, research integrity concerns about extremely high publication rates are real and well documented. Ioannidis and colleagues identified scientists who publish at volumes that make it implausible for a single individual to meaningfully supervise all elements of study design, data analysis, manuscript preparation, and peer review [8]. This work does not portray skepticism as envy but instead frames it as a legitimate inquiry into oversight, authorship practices, and the risk of insufficient review. Studies of retractions and authorship irregularities underscore the need for careful monitoring of contributorship and quality assurance in high-volume research environments [9,10]. These concerns must be taken seriously, not minimized or dismissed.

Why institutions struggle to evaluate outliers

Institutions often struggle to evaluate highly productive faculty because conventional metrics are insufficient to capture mentorship, team leadership, or contributions to rigor and reproducibility (Table 2). Overreliance on volume-based metrics risks undervaluing clinician-educators, systems innovators, and community-engaged scholars. Conversely, reputational dependence on a few highly productive individuals can create vulnerability if their workload becomes unsustainable or if integrity concerns arise. In many settings, high-performing faculty are assigned disproportionate administrative responsibilities, a practice that can dilute research focus, accelerate burnout, and undermine the very productivity that institutions value. Furthermore, resources such as protected time, research assistants, and discretionary funding may concentrate around already advantaged groups unless institutions proactively audit and monitor equity in allocation [11-13].

Differentiated excellence as a path forward and institutional safeguards for high-volume scholarship

A more balanced and modern approach is one of differentiated excellence. Academic medicine benefits from multiple pathways for contribution: the research-intensive investigator who drives scientific innovation; the clinician-educator who shapes the next generation of practitioners; the clinician-scientist who bridges laboratory and bedside; and the systems innovator who improves quality, safety, and equity of care. Each pathway requires distinct skills, opportunities, and forms of evaluation. A differentiated model does not elevate one pathway above others but instead articulates clear expectations, transparent criteria, and equitable access to resources. Within such a framework, high productivity becomes one valued form of excellence rather than the dominant or default one. Safeguards are essential to supporting outliers responsibly. Transparent contributorship statements, such as the CRediT taxonomy, help delineate each author’s intellectual role and prevent honorary authorship. Field-normalized and fractional authorship metrics provide more accurate assessments of scholarly contribution than raw publication counts. Institutions can adopt expectations that senior authors document supervisory processes for data integrity and analysis. Random or targeted reproducibility audits can protect both scientific rigor and the reputational risk borne by high-volume authors [14]. Research-management infrastructure, including analysts, statisticians, and project managers, can ensure adequate oversight and reduce the burden on senior investigators. Finally, regular audits of resource distribution by gender, race, and ethnicity, and rank can prevent differentiated tracks from perpetuating historical inequities [12] (Table 2).

**Table 2:** Institutional challenges and corresponding strategies for supporting diverse excellence

Institutional challenge	Description	Recommended institutional strategies
Misaligned metrics	Evaluation systems emphasize volume over originality, rigor, or non-research contributions.	Implement differentiated excellence tracks (research-intensive, clinician-educator, clinician-scientist, systems innovator) with pathway-specific evaluation criteria.
Integrity and oversight concerns	Extreme productivity can strain authorship oversight, data review, and methodological supervision.	Require transparent contributorship (e.g., CRediT taxonomy), implement reproducibility checks, document senior-author oversight processes, and use field-normalized and fractional authorship metrics.
Administrative overload	Highly productive faculty may be disproportionately assigned administrative, educational, or service responsibilities.	Provide protected time, administrative relief, and structured research-support infrastructure (project managers, statisticians, analysts).
Equity and resource-allocation issues	Accumulated advantages in protected time, funding, and staffing can unintentionally exacerbate inequities across gender, race, ethnicity, or rank.	Conduct annual audits of resource distribution, ensure transparent criteria for allocating protected time and support, and monitor for disparities through structured institutional review.

Supporting excellence while preserving integrity

High-performing faculty can serve as catalytic assets to their departments when supported appropriately and evaluated fairly. Their productivity can accelerate discovery, attract motivated trainees, and elevate institutional prestige. However, responsible stewardship requires institutions to embed safeguards that protect scientific quality, ensure fair attribution, and distribute resources transparently. At the same time, institutions must recognize that excellence in academic medicine is multidimensional. Clinical mastery, educational innovation, community engagement, and systems improvement are just as essential to the academic mission as research productivity. Outliers do not threaten academic medicine; they reveal the full span of its performance variability. What threatens the field is an evaluation culture that undervalues meaningful contributions that fall outside narrow productivity metrics or that fails to attend to the integrity, equity, and sustainability of high-volume scholarship. A balanced framework-one that embraces differentiated excellence while safeguarding rigor and fairness-can help academic medicine support all faculty in ways that advance science, education, and patient care.
